# Solution-phase synthesis and characterization of alkaline earth polysulfides as colloidal nanocrystals

**DOI:** 10.1039/d5na00587f

**Published:** 2025-10-02

**Authors:** Daniel C. Hayes, Omair Z. Choudhry, Shubhanhsu Agarwal, Kiruba Catherine Vincent, Huamã Belmonte, Rakesh Agrawal

**Affiliations:** a Davidson School of Chemical Engineering, Purdue University West Lafayette IN 47907 USA agrawalr@purdue.edu; b Chemical Engineering, Escola Politécnica da Universidade de São Paulo São Paulo Brazil

## Abstract

Solution-chemistry fabrication of semiconductor materials is an attractive synthesis method that allows for easy post-synthesis use in various applications. In this work, we investigate the solution-phase synthesis of a lesser-studied class of semiconductor materials, the binary sulfides of alkaline-earth (AE) metals and their potential for forming polysulfides. Studies have shown that metal polysulfides are widely applied as cathode materials in metal–sulfur batteries and isolated metal polysulfides outside of sulfur-containing solutions are quite rare. Other studies have shown that this material system has the potential to be a wide-bandgap semiconductor or superconducting electride and can also be used as an AES_*n*_ precursor to access certain AE-M-S ternary materials. We show that the synthesis of Ba and Sr polysulfides is strongly correlated to the reaction temperature and that the length of the S_*n*_^2−^ oligomer chain is the dependent variable. To the best of our knowledge, we also report the synthesis of a previously unreported polymorph of SrS_2_. With bandgaps estimated *via* UV-vis spectroscopy, spanning the upper energy range of the visible spectrum (2.4–3.0 eV), the AE polysulfides have potential for semiconducting applications, such as displays, transparent conducting oxides, or tandem photovoltaics, among others. Paired with their high crystal abundance and relatively low toxicity, these materials make good candidates for future studies as wide-bandgap semiconductors.

## Abstract 2

Using just three precursors (oleylamine, alkaline-earth acetylacetonates, and elemental sulfur), syntheiszed alkaline-earth polysulfide nanocrystals can be tuned *via* changes to reaction temperature.

## Graphical abstract

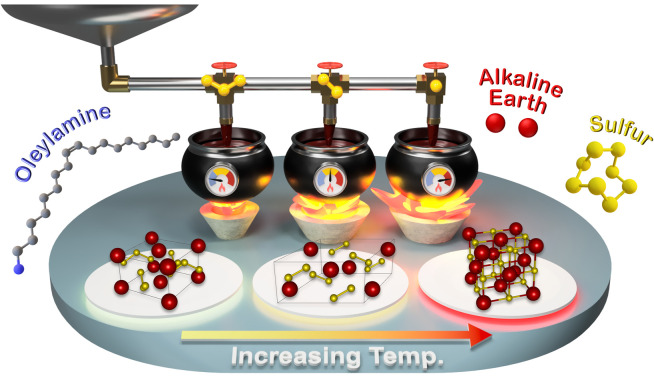

## 1. Introduction

Chalcogenide semiconductors are a class of materials that cover an enormous array of different compositions, properties, and applications, including photovoltaics, thermoelectrics, light-emitting diodes (LEDs), and other devices that take advantage of the unique optoelectronic properties offered by these materials. Many iterations of chalcogenide semiconductors—from the likes of Cu(In,Ga)(S,Se)_2_, Cu_2_-II-IV-(S,Se)_4_ (II = Sr, Ba, Zn, Cd, for example; IV = Sn or Ge for example), and II–VI and IV–VI chalcogenides (*i.e.* CdSe or PbS), among many others—have been extensively studied over the years and have seen considerable success as materials for photovoltaic applications or LEDs.^1–14^ For applications in which a wider band gap is desirable (*i.e.* as buffer layers in solar cells, transparent thin film transistors, photoelectrocatalysts for water splitting, and visible LED displays, among others), alkaline-earth (AE)-based chalcogenides have begun to receive considerable attention in binary and other multinary materials.^15^ This includes chalcogenide perovskites,^16,17^ noteworthy for having received considerable attention in the last decade for having an extremely high absorption coefficient (>2 × 10^5^ cm^−1^ at *E* = *E*_g_ + 0.5 eV for BaZrS_3_)^18^ and an increased, across-the-board stability over halide perovskites,^19,20^ with various studies of its fundamental properties^21–25^ and the moderation of synthesis protocols for increased ease-of-use.^26–32^ Aside from being earth-abundant, AE-based chalcogenides can also be used as alternatives to other heavy metal chalcogenides, such as many of the popular II–VI and IV–VI compounds containing Cd or Pb, which carry concerns related to toxicity.

Another unique property of chalcogens is their ability to stabilize into polychalcogenide chains of various lengths (X_*n*_^2−^, *n* ≥ 2). These polychalcogenide chains possess unique chemical attributes and can be used to access kinetically stabilized or metastable materials, many of which may possess attractive and unusual chemical and physical properties.^33^ Polysulfides, in particular, see extensive use in metal–sulfur batteries, particularly for the Li–S system.^34^ Despite the advances made in Li–S batteries recently, challenges remain, such as their propensity for forming dendrites during cell operation and the anticipated cost increase and ever-limited availability of Li. This has motivated researchers to investigate other metal–sulfur systems, including those of Mg and Ca as alternatives to Li,^35^ potentially opening the door to further research into other AE-sulfur battery systems.

**Fig. 1:**
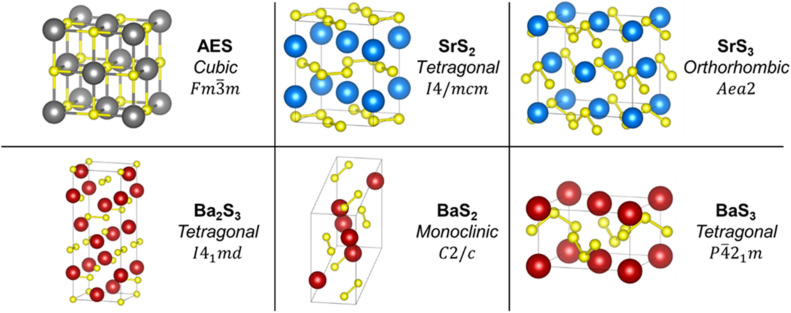
Unit cells of the standardized AE polysulfide crystal structures of the ICSD and the *Fm*3̄*m* rock salt structure of AE monosulfides (excluding BeS). Each illustration is listed with its chemical formula, crystal system, and space group to which it belongs. Sr atoms are in blue, Ba atoms are in red, S atoms are in yellow, and any AE atom (from Mg to Ba) is in grey. The collection codes used for generating these illustrations are ICSD# 642 (SrS_2_), ICSD# 23638 (SrS_3_), ICSD# 70058 (Ba_2_S_3_), ICSD# 2004 (BaS_2_), and ICSD# 70059 (BaS_3_). These illustrations were generated using VESTA.^70^

Over the years, the scientific community has seen an increasing number of works on AE polychalcogenides, ranging from fundamental studies on their chemical and physical properties^36–42^ to their potential for applications in devices, including thermoelectric devices, photovoltaic devices and photodetectors.^43–46^ Among the AE metals, polysulfides of Mg up to Ba have been reported, but crystal structures have been standardized only for those of Ba and Sr,^47^ possibly owing to the difficulty in synthesizing quality and/or isolating stable samples of the lighter AE polysulfides for a detailed characterization. Fig. 1 shows the crystal structure of these standardized AE polysulfides, namely SrS_2_, SrS_3_, BaS_2_, BaS_3_, and Ba_2_S_3_, a non-integer polysulfide, along with the *Fm*3̄*m* rock salt structure of the AE monosulfides (excluding BeS, which crystallizes in the *F*4̄3*m* zinc blende structure), which are part of the Inorganic Crystal Structure Database (ICSD). Notably, the polysulfide chains containing S–S bonds are seen in these structures with bent S_3_^2−^ chains for the trisulfides. Previous work shows that the AE polysulfides, BaS_2_ and BaS_3_, have unique chemical and electronic structures that allow for accessing more complex ternary systems *via* solid-state techniques under milder conditions.^40^ Various methods are used to synthesize metal chalcogenides, including solid-state techniques, but solution-based methods—of particular interest for this work—have also been explored. Synthesis and fabrication of materials *via* solution methods can bypass the need for capital-intensive and/or energy-demanding equipment (*i.e.*, vacuum-based methods, such as sputtering, atomic-layer deposition, or molecular beam epitaxy and/or high-temperature furnaces for solid-state reactions) and can additionally allow for easy post-synthesis use in deposition or thin film fabrication.^3,48–51^

In this work, we studied the controlled formation of AE polysulfides, particularly of Ba and Sr, *via* solution-based methods. We demonstrate that the formation of these materials is highly dependent on reaction temperature, resulting in polysulfides of different S_*n*_^2−^ chain lengths (*n* = 2–3). Temperature has been known to be a key factor in dictating the polychalcogenide chain length due to the thermal instability exhibited by X_*n*_^2−^ chains,^33^ with lower temperatures enabling predominantly larger *n* values and higher temperatures enabling predominantly smaller *n* values.^52^ In an alkylamine–sulfur system, used in our synthesis protocol, polysulfide anions are formed upon a reactive dissolution of elemental sulfur with the alkylamine, and are expected to represent one of the primary reactive sulfur species in solution.^53^ If we assume a similar trend of polysulfide chain length as a function of temperature in the amine–sulfur system, it would seem that the selective synthesis of metal polysulfides of varying chain lengths should be possible *via* solution-based methods. By tuning the reaction temperature in our system, we can access BaS_2_, BaS_3_, and, to the best of our knowledge, a previously unreported polymorph of SrS_2_ by reacting AE salts with elemental sulfur in oleylamine. We show that the chain length, *n*, of the synthesized AE polysulfides follows a trend where higher temperatures yield shorter chain lengths (lower *n* values) down to *n* = 1, while lower temperatures can be used to access polysulfides. We show that with our methods here, barium has the most control over chain length, between 2 ≤ *n* ≤ 3, while strontium is only able to stabilize the *n* = 2 polysulfide. Ca was also included in this study, but was found to form only the monosulfide (CaS) regardless of the reaction conditions used during this study. In addition to the synthesis of these polysulfide materials, we also determine the absorption properties of these materials *via* UV-vis and assess their viability for use in optoelectronic devices.

## 2. Experimental methods

### 2.1 Materials

Barium acetylacetonate hydrate (Ba(acac)_2_·*x*H_2_O) and strontium acetylacetonate hydrate (99%, Sr(acac)_2_·*x*H_2_O) were purchased from Strem. Calcium acetylacetonate hydrate (99.95%, Ca(acac)_2_·*x*H_2_O), elemental sulfur (≥99.99% trace metals basis, S), calcium hydride (reagent grade, 95%, CaH_2_), and oleylamine (technical grade, 70%, OLA) were purchased from Sigma-Aldrich. The AE acac salts were placed in a round-bottom flask and dried *in vacuo* for ∼12–16 hours using Schlenk line techniques with a heating mantle set to 150 °C. The oleylamine underwent successive freeze–pump–thaw cycles, followed by stirring for ∼12–16 hours over CaH_2_*in vacuo* at room temperature, followed by an additional 2 hours at a heating mantle setpoint of 200 °C to remove residual water while still *in vacuo*. After drying over CaH_2_, the oleylamine was decanted and distilled *in vacuo*, followed by storage over 3 Å molecular sieves. The S and CaH_2_ were used as received.

### 2.2 Nanocrystal synthesis

All material preparation and post-synthesis workup were performed in an inert, N_2_-filled glovebox. For the synthesis of AE sulfide nanocrystals, our standard procedure used a metal : S ratio of 1 : 12. In a typical synthesis, 0.25 mmol of the AE salt was combined with 3 mmol of S flakes in 3 mL of dried OLA into a borosilicate glass microwave reaction vial with a small PTFE-coated magnetic stir bar. Unless otherwise specified, the reactions were performed in a Biotage Initiator EXP or Biotage Initiator+ 400 W microwave reactor for 60 min at varying temperatures from 100 °C to 300 °C at a stir rate of 600 rpm. After the synthesis, nanocrystals were washed using a ∼1 : 5 mixture of toluene : IPA—first adding toluene and mixing, followed by IPA and mixing—and centrifuged at 14 000 rpm for 5 min. This washing procedure was repeated one additional time before storing the synthesized nanocrystals in a scintillation vial with toluene in the glovebox before further usage and characterization.

### 2.3 Characterization

Raman spectra were collected using a Horiba/Jobin-Yvon HR800 Raman spectrometer with a 632.8 nm wavelength excitation laser. Powder X-ray diffraction (pXRD) data were collected using a Rigaku SmartLab diffractometer with a Cu Kα (*λ* = 1.5406 Å) source operated at 40 kV/44 mA in parallel-beam mode. When identifying experimental pXRD data, standardized data from the Inorganic Crystal Structure Database (ICSD) were used as a reference. Elemental composition measurements were obtained using a Fisher XAN 250 X-ray fluorescence (XRF) instrument at a 50 kV voltage, equipped with a silicon drift detector, primary nickel filter, and flowing helium gas purge. Transmission electron microscopy (TEM) images were obtained using both Tecnai G2 20 and Talos 200i microscopes with an accelerating voltage of 200 kV. UV-vis diffuse reflectance data were collected from nanocrystal drop-cast films on soda-lime glass substrates using a PerkinElmer Lambda 950 spectrometer equipped with an integrating sphere. The reflectance spectra were then transformed to absorption spectra (wavelength *vs.* absorbance) using the Kubelka–Munk function to estimate the band gap.

## 3. Results and discussion

To minimize experimental complexity, our primary synthesis protocol of the AE polysulfides consisted of a simple system with only three components: a solvent/ligand (OLA), sulfur source (elemental sulfur), and an AE source (AE acac salts). In its elemental form, sulfur can exist as many different allotropes, but thermodynamically favors a crown-shaped, cyclooctasulfur (S_8_) ring as its most stable form—important to consider for understanding how these polysulfides might form. The number of S units in this more stable form can consequently decrease or increase in the liquid or gaseous state, which is caused by changes in temperature and/or pressure due to the sensitivity of polychalcogenides to changes in these variables.^33,52,54^ For sulfur in solution with primary amines, the behavior and chain length of elemental sulfur is less understood, but it is known that reactive dissolution does generate polysulfide anions *via* a ring-opening mechanism.^53^

**Fig. 2:**
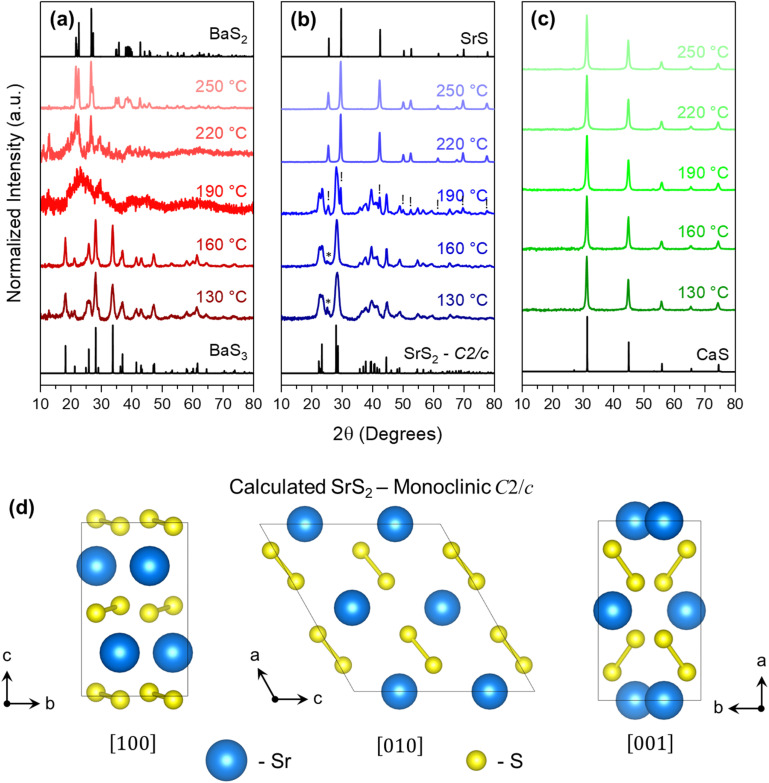
Temperature studies of the Ba-, Sr-, and Ca-S systems for the temperature range of 130 °C to 250 °C, characterized *via* pXRD, in (a)–(c), respectively. We attribute the peaks marked with ! and * in the Sr–S system to SrS and traces of SrS_3_, respectively. The standards used in this figure are ICSD# 70059 (BaS_3_), ICSD# 2004 (BaS_2_), ICSD# 28900 (SrS), and ICSD# 28902 (CaS). Shown in (d) is the unit cell of the monoclinic *C*2/*c* crystal structure of SrS_2_ from three different orientations, determined and optimized during Rietveld refinement of the 160 °C experimental data of the Sr–S system by adjusting the BaS_2_ standard to best fit the Sr–S data. More information can be found in the supporting information in Fig. S2 and Table S1. The crystal structure illustration in (d) was generated using VESTA.^70^

The structural characterization of polysulfide nanocrystals formed *via* solvothermal reactions in a microwave reactor is shown in Fig. 2, along with data obtained *via* pXRD measurements. Data is shown for the temperature studies of the Ba-, Sr-, and Ca-S systems in the range of 130 °C to 250 °C, which exemplifies the effect of temperature on polysulfide formation in alkylamine–sulfur solutions, particularly for the Ba–S and Sr–S systems. Beginning with the Ba–S system, we observe a good agreement between the lower-temperature products (130–160 °C) and the BaS_3_ standard. When increasing the temperature above 160 °C but below 250 °C, the pXRD results seem to indicate that a “region of instability” may exist, preventing the formation of either of the expected Ba polysulfide species, especially for the 190 °C reaction product. Favorability towards BaS_2_ is observed at 220 °C, but it is currently unclear why this temperature region does not instead primarily show a mixture of BaS_3_ and BaS_2_ products. Once the reaction temperature is raised to 250 °C, there is again good agreement with previous standards—BaS_2_ in this case. Additional data on the reactions of the Ba–S system performed at 100 °C and 300 °C are given in Fig. S1, where it is observed that the reaction pressure and/or the residual presence of volatile reaction byproducts can also influence the reaction product. The close match of the Ba-polysulfide spectra with the ICSD standards indicates the degree of phase purity achievable for these synthesis protocols.

**Fig. 3:**
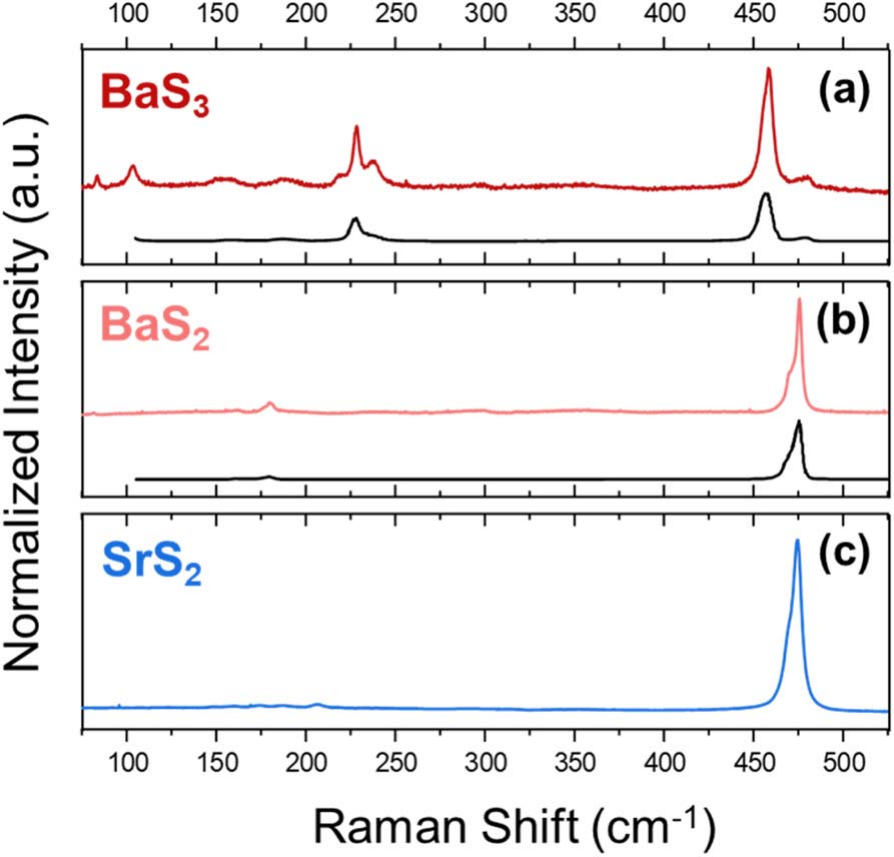
Raman spectroscopy of the alkaline-earth polysulfide samples of BaS_3_ (a), BaS_2_ (b), and SrS_2_ (c), at 160 °C, 250 °C, and 160 °C, respectively, in a microwave reactor. The plots in black at the bottom of (a) and (b) were digitized from Sasaki *et al.* and are used for visual comparison of BaS_3_ and BaS_2_, respectively, to previously reported work.^40^

Next, the Sr–S system also shows tunability for its polysulfide chain length but is shown here to exist only as the disulfide or monosulfide. SrS_3_ was not obtainable as a phase-pure product *via* the methods explored in this work. Analyzing in detail the 130 °C and 160 °C reaction products, though, there is an indication that it may appear as a minor product for the Sr–S reactions performed at lower temperatures. Its formation may suggest that, through further optimization of reaction parameters, phase-pure SrS_3_ nanocrystals may be attainable. The most notable result from the Sr–S system is the formation of, to the best of our knowledge, an unreported polymorph of SrS_2_ in the range of 130 °C to 190 °C. This form of SrS_2_ exhibits structural characteristics similar to those of BaS_2_ based on qualitative similarities in the pXRD patterns and Raman spectra (shown in Fig. 3).

A search through the ICSD and the International Centre for Diffraction Data (ICDD) reveals that these databases do not contain any crystal structures in the Sr–S family that closely match our experimental SrS_2_ data. To further justify our claim that this is a new polymorph, closely related in structure to the monoclinic *C*2/*c* phase of BaS_2_, we performed a Rietveld refinement on the experimental data using a simulated structure of SrS_2_ in this same crystal motif. This result is shown in Fig. S2 and shows good agreement with the calculated structure. Some additional data on the crystal structure generated during the refinements are given in Table S1 and comparisons between the calculated SrS_2_ structure and other structures in the ICSD are also provided in Fig. S3 for the readers' reference.

Analyzing the Raman spectra, we see a striking similarity between that of the BaS_2_ and SrS_2_ nanocrystals, with the most prominent peak for both located at ∼474 cm^−1^ and a similar peak shape with (at least) two overlapping signals from the stretching signal of the S_2_^2−^ bonds. The Raman spectra obtained for polysulfides similar to these materials are dominated by covalent bonds in the S_*n*_^2−^ chains,^36^ which supports the notion that they reside in similar coordination environments and thus have a similar overall crystal structure. It should be noted that previous studies on Sr polysulfides were carried out *via* solid-state methods. One such study conducted the synthesis at a high temperature and pressure (900 °C, 20 kbar) using SrS and elemental S to generate SrS_2_ crystals.^55^ Another study investigated the reactions of Sr(OH)_2_ with elemental sulfur at relatively low temperatures (200 °C), presumably at atmospheric pressures under a flow of N_2_ gas (though the study does not make it entirely clear), to form SrS_3_, followed by decomposition at 300 °C, breaking down the S_3_^2−^ chains, to generate SrS_2_.^56^ These studies may indicate that either high pressures are needed to fabricate polysulfides of Sr, or that certain precursors (*i.e.* Sr(OH)_2_) may be needed to provide a kinetic pathway for the formation of Sr polysulfides. For reactions starting at 190 °C and above, we observe the formation of SrS alongside SrS_2_ at 190 °C and phase-pure SrS at temperatures above 220 °C.

In addition to the structural characterizations shown thus far, XRF measurements revealed a Ba : S ratio of ∼1 : 3 and ∼1 : 2 for the BaS_3_ and BaS_2_ products, respectively, and a Sr : S ratio of ∼1 : 2 for the SrS_2_ product. Plots of these data are shown in Fig. S4. The Ca–S system was also studied for its potential to form polysulfides *via* solution-phase methods. As stated previously, this material system has been used in Ca–S batteries^35^ and is also predicted to have some interesting electronic properties as superconducting electrides; however, to date, it has only been computationally predicted and experimentally isolated as polysulfides under high pressures.^37,43^ Over the entire temperature range used in this study (130–250 °C), only the monosulfide, CaS, was found to form. Powder XRD characterization data for the Ca–S experiments are also shown in Fig. 2c, with additional analysis of the pXRD data in Fig. S5. Here, it is shown that the growth and ultimate size of the CaS nanocrystals are not particularly sensitive to changes in temperature (at least given the conditions and temperature range investigated here), suggesting that, given the precursor choices in this study, nucleation and growth occur quite rapidly. The general sharpness of the pXRD peaks further corroborates this claim.

Based on these results, the polysulfide anion chain length in the isolated crystal structure is not solely temperature dependent, as the AE cation is also shown to affect the chain length of the isolated product. Under our reaction conditions at 160 °C, for example, the majority product with Ba, Sr, and Ca is with *n* = 3, 2, and 1, respectively. One might consider cation size to play a prominent role, but considering another example in PbS nanocrystals, it is shown that with the use of sulfur–oleylamine solutions at temperatures as low as 40 °C, only the monosulfide of Pb is formed.^57^ The ionic radius of Pb^2+^ is comparable to Ba^2+^ and Sr^2+^ (in pm, 133, 149, and 132, respectively), indicating that there are likely other contributing factors. This phenomenon likely stems from the relatively unstable thermodynamics and (due to their similar Gibbs free energies) the complex disproportionation/comproportionation equilibria present with polysulfide anions of different *n* values, resulting in very few metal polysulfides reported to be isolatable outside of mixtures with excess sulfur.^34,58^ Various techniques exist to characterize polysulfide species,^58^ but the complex equilibria of these polysulfides make it quite difficult to characterize individual species, which is beyond the scope of this work.

**Fig. 4:**
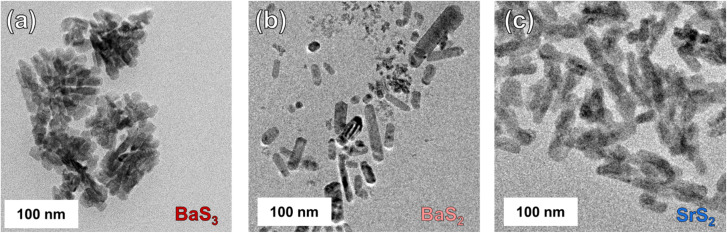
TEM images of BaS_3_, BaS_2_, and SrS_2_ nanocrystals are shown in (a)–(c), respectively. These nanocrystals were synthesized at 160 °C, 250 °C, and 160 °C in a sealed microwave vessel for BaS_3_, BaS_2_, and SrS_2_, respectively. The nanocrystals synthesized in this study appear to all resemble shape anisotropy with rod-like structures.

TEM data are shown in Fig. 4, S6 and S7 for the three species with both low-resolution and high-resolution images, respectively, for gauging size dispersity and observing crystal planes of the nanocrystals. Indicated by the general sharpness of the pXRD data (Fig. 2) and shown in the TEM images (Fig. 4), we can see that nanocrystal sizes are on the order of (at least) several tens of nanometers. Nanocrystal size can play a big role in the colloidal stability of nanomaterials by overcoming Brownian motion in solution once the nanocrystals surpass a critical size. As such, we at least partially associate the observation that these nanocrystals settle out of solution after only a few minutes of resting due to their larger crystal sizes. Another possible significant contribution is the surface functionality of the synthesized nanocrystals, which dictates ligand coverage and aggregation potential. OLA, as used in this protocol, is widely used in a variety of nanocrystal syntheses as a stabilizing ligand for generating colloidally stable nanomaterials. In the case of these materials, it seems as though OLA alone is not sufficient for adequately stabilizing them. This may be due to a lower binding affinity of the –NH_2_ functional group to the surfaces of the AE polysulfides.

Apart from this discussion on surface functionality, we can see that each nanocrystal species presents a moderate degree of shape anisotropy. All species appear to have somewhat similar rod-like morphologies, with BaS_3_ appearing to form as feather- or fan-like clusters. Further work with these synthesis protocols, including experimentation with different ligands (*i.e.*, carboxylic acids, phosphines, thiols, *etc.*) and different precursors (though care should be taken to avoid insoluble AE precursors or less reactive precursors such as the AE halides^59^) would be needed to synthesize more colloidally stable nanocrystals—important for downstream usage for homogeneous inks and/or coatings. More discussion on precursor choices is given in Fig. S8, along with the supplementary discussion that follows.

**Fig. 5:**
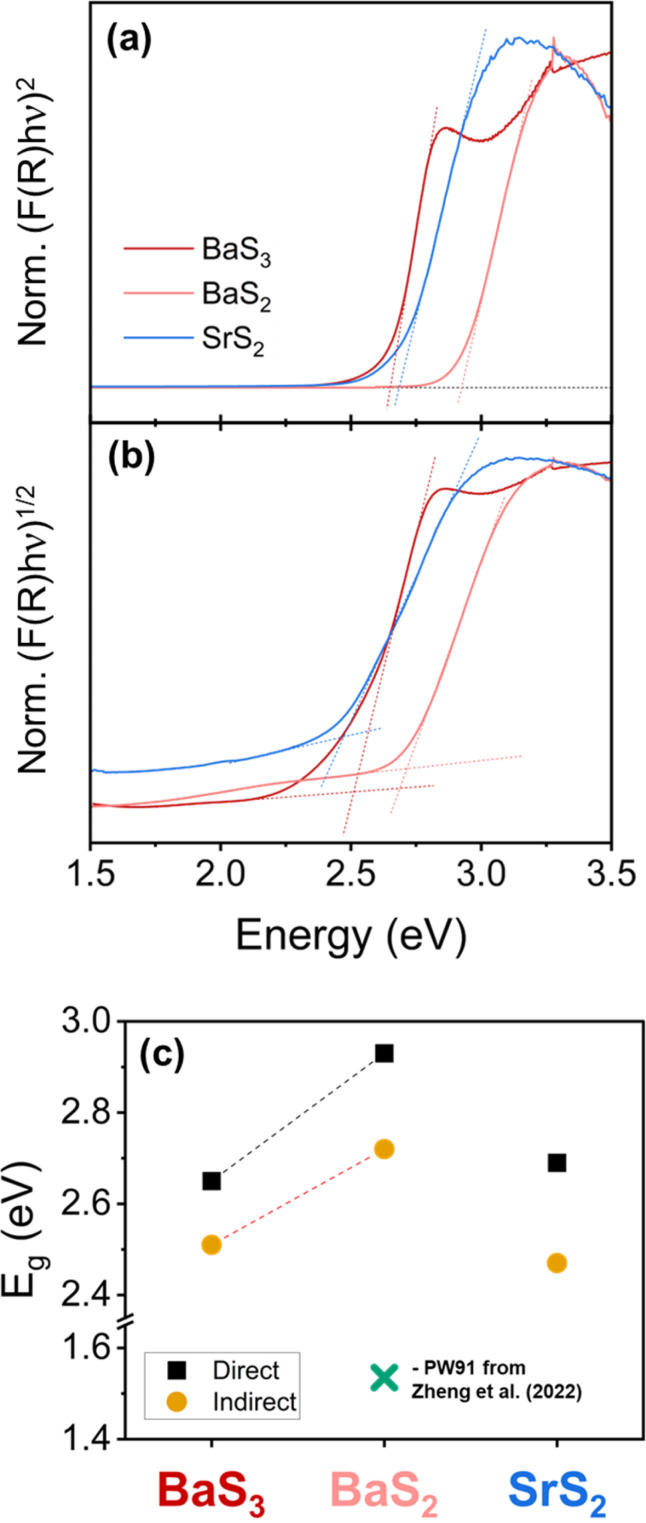
Kubelka–Munk transformations used to determine the bandgap of the synthesized nanocrystals. The direct transformation is shown in (a), and the indirect transformation is shown in (b). (c) Displays the bandgaps determined from these measurements with the direct and indirect energy gaps of BaS_3_, BaS_2_, and SrS_2_ being 2.65, 2.51; 2.93, 2.72; and 2.69, 2.47 eV, respectively. The blue cross in (c) represents the calculated data from the computational study on Ba dichalcogenides.^46^ These measurements were performed on nanocrystals synthesized at 160 °C, 250 °C, and 160 °C for BaS_3_, BaS_2_, and SrS_2_, respectively.

To our knowledge, only one study thus far has been reported for the band structure of any of the compounds synthesized in this work—specifically, monoclinic BaS_2_—via density functional theory calculations using the generalized gradient approximation with the PW91 exchange–correlation (XC) functional.^46^ In this previous work, BaS_2_ is predicted to have an indirect bandgap of 1.535 eV and a direct bandgap transition at a slightly higher energy. The PW91 functional tends to underestimate the bandgap of semiconductor materials, as opposed to more reliable XC functionals for band structure analysis, such as the HSE06 or mBJ functionals,^60–64^ which may explain the deviation between the theoretical data and our experimental data. The experimental bandgaps were estimated using the Tauc method (*α*·*hν*)^1/*γ*^ = *B*(*hν* − *E*_g_), where *γ* = 1/2 for direct and 2 for indirect bandgaps. Since we performed reflectance measurements, the Kubelka–Munk function, *F*(*R*), is applied in place of absorption, *α*.^65^ The literature on the bandgap of these materials is limited, so we have reported both direct and indirect band gaps in Fig. 5. The data presented has been normalized to the maximum of [*F*(*R*)·*hν*]^1/*γ*^ in the window shown so that the shape and position relative to the energy of each curve can be compared with one another. For all three species, we see a significant absorption onset characterizing the bandgap of each material. BaS_3_ and SrS_2_ have similar bandgaps of around 2.5–2.7 eV, while BaS_2_ has the highest bandgap among these three materials, approaching 3 eV for a direct bandgap estimation. A bandgap increase from BaS_3_ to BaS_2_ is arguably as expected, considering the band gap for BaS is reported to be in the range of 3.5–3.9 eV.^42^ Additionally, BaS_3_ appears to show a second absorption event at higher energies, possibly due to a separate energy transition requiring energies slightly higher than the main energy transition. Raw data of these measurements plotted as % reflectance *vs.* wavelength is also provided in Fig. S9.

We should also note that in their native state, these materials did not show any distinct photoluminescence (PL) when cast as a film. If we assume these are direct bandgap materials, this could possibly be indicative of a defective material, but in nanocrystals, deleterious surface states must also be considered for the lack of notable PL. Since the properties of nanocrystals are largely dominated by their surfaces, an accumulation of harmful surface defects caused by dangling bonds from improper or incomplete surface passivation can easily quench any PL signal.^66^ This can be addressed by fabricating core–shell nanocrystal structures where the material of interest resides in the core, and a passivating shell is formed around the core to promote increased PL yields.^67^ Additionally, ligand exchange procedures can be used to replace the native ligands from the nanocrystal synthesis with a ligand(s) better suited to passivate harmful surface defects.

## 4. Conclusions

The earth-abundant, AE polysulfides are a lesser-studied class of materials, but are predicted to have some interesting attributes, making them attractive for semiconducting applications. This study investigates the solution-based synthesis of three different AE polysulfide nanocrystals (BaS_3_, BaS_2_, and SrS_2_) *via* colloidal methods by the variation of one key parameter—the reaction temperature. Given a heavier AE metal (Ba or Sr), changing the reaction temperature allows us to fine-tune the chain length of the S_*n*_^2−^ chains, resulting in the different species discussed. In addition to the synthesis of Ba polysulfides, we discuss the formation of, to the best of our knowledge, an unreported polymorph of SrS_2_, which is different from that reported in the Inorganic Crystal Structure Database for SrS_2_. We then assess some of the basic absorption properties of these materials *via* UV-vis measurements and find that their bandgaps fall within the range corresponding to wide-bandgap semiconductors.

Considering the instabilities of the polysulfides and the protocols in this study unable to stabilize certain phase-pure polysulfide species (*n* > 2 for Sr, *n* > 1 for Ca and other non-integer values of *n* such as with Ba_2_S_3_), it should be recalled that solution-based nanomaterials have the ability to access metastable phases as a result of non-equilibrium reaction kinetics and the influence of surface energies on the system thermodynamics. Additionally, cation-exchange methods have often been performed to access metastable polymorphs of various materials.^68,69^ With this in mind, with the right precursors, solvents, ligands, and other reaction parameters, stabilization of these additional phases may be possible. Further work is also required to increase the colloidal stability of these materials, as flocculation observed during sample handling suggests aggregation in their native form. Additionally, photoluminescence of these materials was not observed; however, surface modifications, including but not limited to core–shell structures and/or ligand exchange methods—which were not investigated in this study—should be explored to assess if deleterious surface states are responsible for the lack of notable photoluminescence. Furthermore, pairing experiments such as these with detailed computational studies on the band structures of these materials would help provide a more thorough idea of their optoelectronic properties. We hope that by expanding the catalogue of synthesizable nanocrystals, this work allows others to explore AE polysulfide nanocrystals and the potential applications that may await these materials.

## Author contributions

DCH: conceptualization, data curation, formal analysis, investigation, methodology, visualization, writing – original draft, writing – review & editing. OC: data curation, formal analysis, investigation, validation. SA: data curation, investigation, validation, writing – review & editing. KCV: data curation, writing – review & editing. HB: data curation, investigation. RA: funding acquisition, supervision, writing – review & editing.

## Conflicts of interest

There are no conflicts to declare.

## Supplementary Material

NA-OLF-D5NA00587F-s001

## Data Availability

Additional data supporting the results in this article have been included as part of the supplementary information (SI), which includes additional pXRD data, XRF data, and TEM data, along with a short supplementary discussion on precursor choices. Supplementary information is available. See DOI: https://doi.org/10.1039/d5na00587f.
