# Supplementary material for: Solution-phase synthesis and characterization of alkaline earth polysulfides as colloidal nanocrystals
Source: Nanoscale Adv. 2025 Oct 2;7(23):7653–62. doi: 10.1039/d5na00587f (PMC12529581; doi:10.1039/d5na00587f)
Supplement: NA-007-D5NA00587F-s001 [file NA-007-D5NA00587F-s001.pdf]

Supplementary Information (SI) for Nanoscale Advances.
This journal is © The Royal Society of Chemistry 2025

Solution-phase Synthesis and Characterization of Alkaline Earth Polysulfides as Colloidal Nanocrystals

Daniel C. Hayes,[a] Omair Choudhry,[a] Shubhanhsu Agarwal,[a] Kiruba Catherine Vincent,[a] Huamã Belmonte,[b] Rakesh Agrawal *[a]

a. Davidson School of Chemical Engineering, Purdue University, West Lafayette, IN 47907, USA

b. Chemical Engineering, Escola Politécnica da Universidade de São Paulo, São Paulo, Brazil

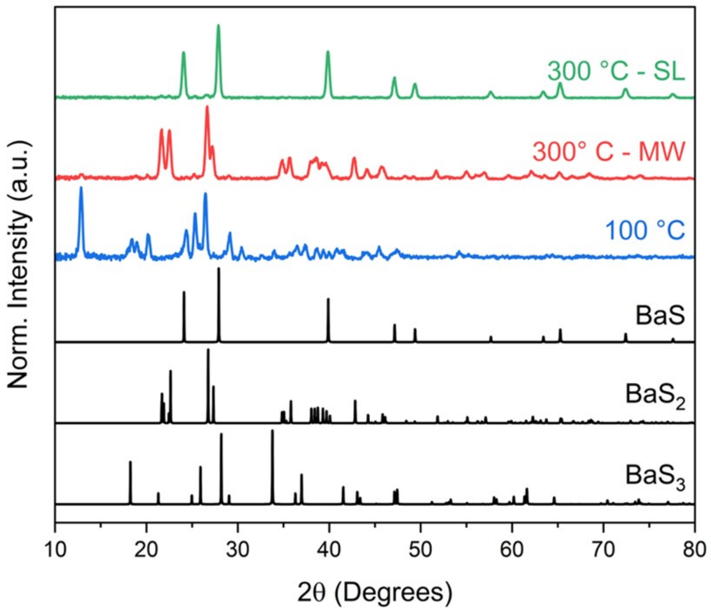

Supporting Information

Figure S1. Additional data to complete the temperature study of the Ba-S system with samples synthesized at 100 °C and 300 °C. The product at 100 °C is not identifiable from any of the known standards of the Ba-S, Ba-O, or Ba-O-S systems. The reaction product at 300 °C is dependent on how the reaction is conducted. In the microwave (MW) reactor at 300 °C with the formation of BaS_2, the reaction takes place in a sealed vessel, keeping any volatile byproducts (i.e. H_2S) in the system and allowing the pressure inside the vessel to increase. In the Schlenk line (SL) reaction* at 300 °C with the formation of BaS, volatile species can escape the system via the SL exhaust, and the pressure is fully dictated by that of the inert (Ar) manifold during operation. In addition to the pressure differences, the rate of heating is much faster in the microwave vial via microwave radiation than the Schlenk line flask (heated via traditional, resistive heating), which could impact the trajectory of relative rates of the various reactions that may be involved, including nucleation. This indicates that in addition to temperature, pressure and/or the residual presence of volatile byproducts and heating rates may play a role in the dominant S_x^{2-} chain length that forms. The pXRD standards used are ICSD# 30240 (BaS), ICSD# 2004 (BaS_2), and ICSD# 70059 (BaS_3).

*The Schlenk line reaction was performed in a 25 mL, 3-neck flask plugged with an *in-situ* thermocouple, Allihn condenser, and a glass plug. Prepared in the glovebox, the flask was sealed and taken to a Schlenk line where the Schlenk line headspace was purged three times via successive vacuum-purge cycles using Ar gas. After purging, the flask was opened to the Schlenk line under Ar flow to begin the reaction.

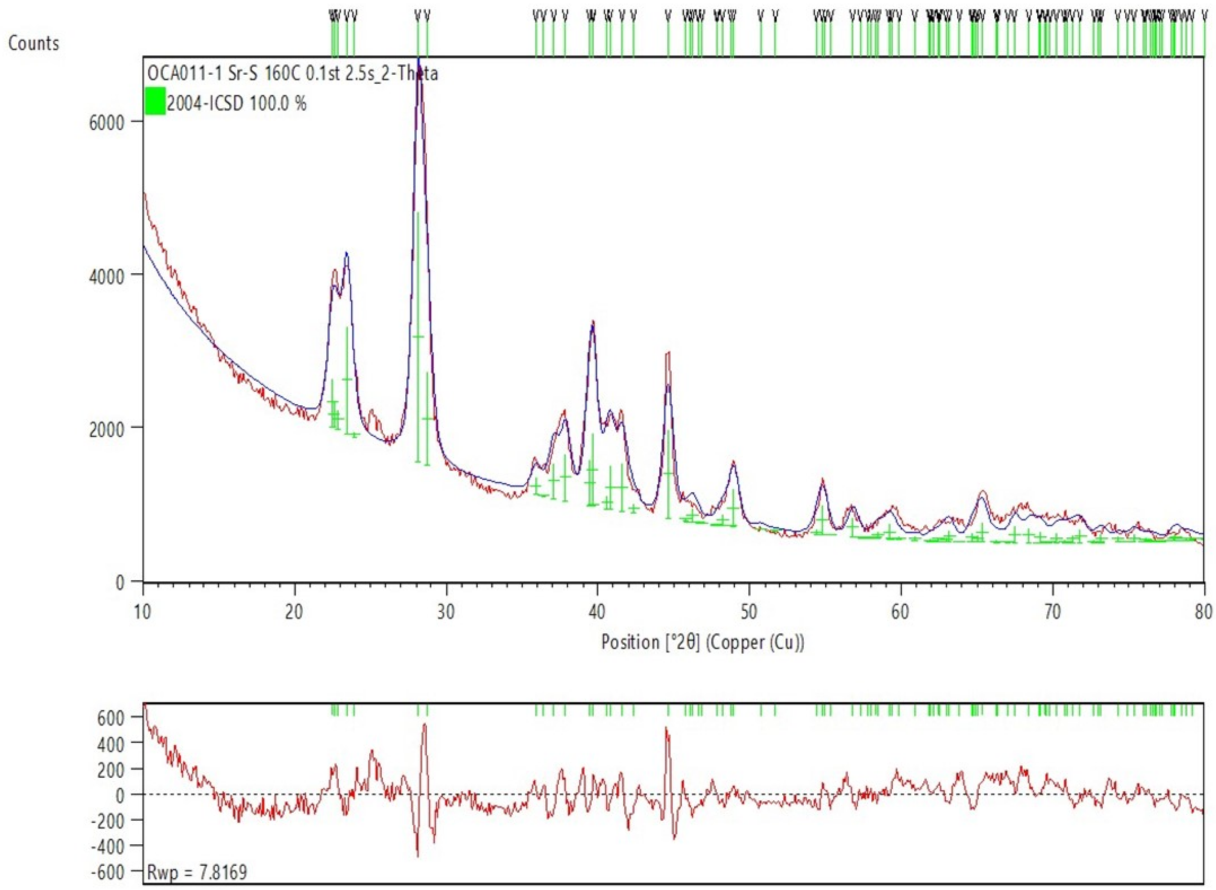

Figure S2. Rietveld refinement of the SrS_2 data (collected from the sample synthesized at 160 °C) using a calculated and optimized structure of SrS_2 based on a starting point of the monoclinic *C2/c* structure of BaS_2 (ICSD# 2004). The fit is shown in the top panel and the residuals in the bottom panel. The refinement shows that this calculated structure matches quite well with that of the experimental data, accounting for some preferred orientation. Additional data of the simulated SrS_2 structure is provided in **Table S1**.

Table S1. Parameters of the Simulated SrS_2 Crystal Structure

<table>
<tr><td colspan="2">Chemical Formula</td><td>SrS_2</td></tr>
<tr><td colspan="2">Crystal System</td><td>Monoclinic</td></tr>
<tr><td colspan="2">Space Group</td><td>C 1 2/c 1 (15)</td></tr>
<tr><td colspan="2">a, b, c [Å]</td><td>9.112(3), 4.544(1), 8.550(3)</td></tr>
<tr><td colspan="2">α, β, γ [°]</td><td>90, 119.403(6), 90</td></tr>
<tr><td colspan="2">V [Å^3]</td><td>308.43430</td></tr>
<tr><td colspan="2">Density (calculated) [g/cm^3]</td><td>3.2673</td></tr>
<tr><td colspan="2">F(000)</td><td>280.0000</td></tr>
<tr><td rowspan="2">Atomic Coordinates</td><td>Sr1 (x/a, y/b, z/c)</td><td>0, 0.359(1), 0.25</td></tr>
<tr><td>S1 (x/a, y/b, z/c)</td><td>0.1574(8), 0.136(3), 0.0215(8)</td></tr>
</table>

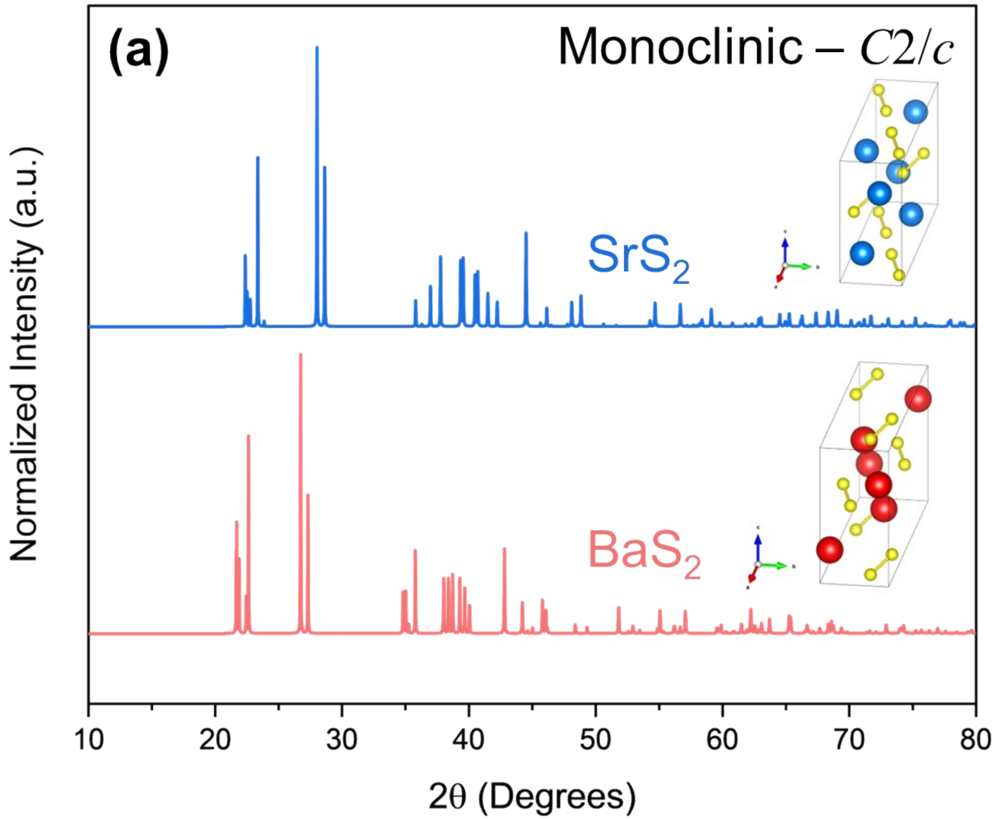

Figure S3. Caption on next page.

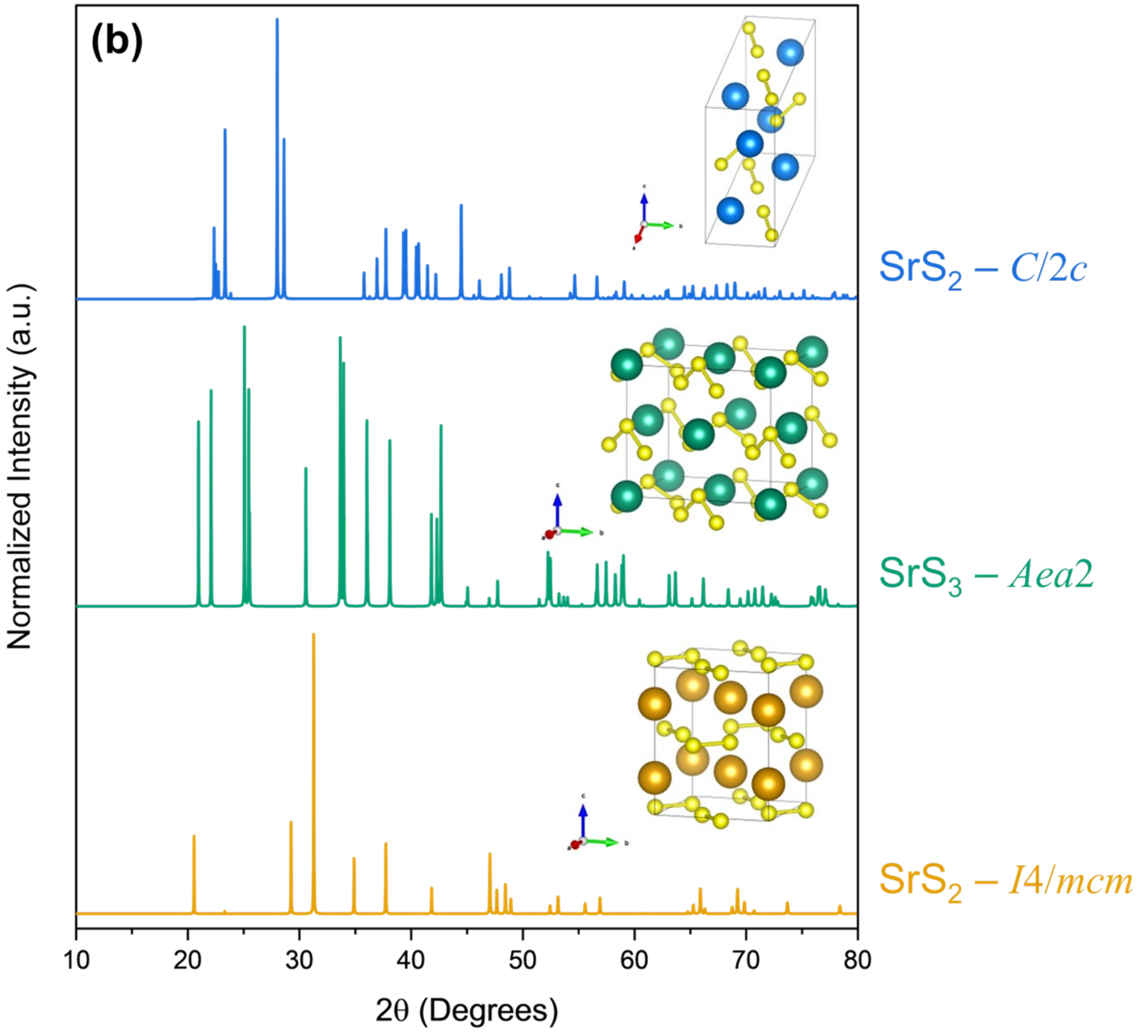

Figure S3. Shown here are comparisons of the standardized crystal structure data of various crystal structures from the ICSD and the simulated SrS_2 – *C*2/*c* structure obtained during Rietveld refinements. (a) shows how the two monoclinic pXRD patterns compare and (b) shows how the simulated SrS_2 pattern compares to the two standardized polysulfides of the Sr-S system in the ICSD. The ICSD standards used here are ICSD# 2004 (BaS_2 – *C*2/*c*), ICSD# 642 (SrS_2 – *I*4/*mcm*), and ICSD# 23638 (SrS_3 – *Aea*2).

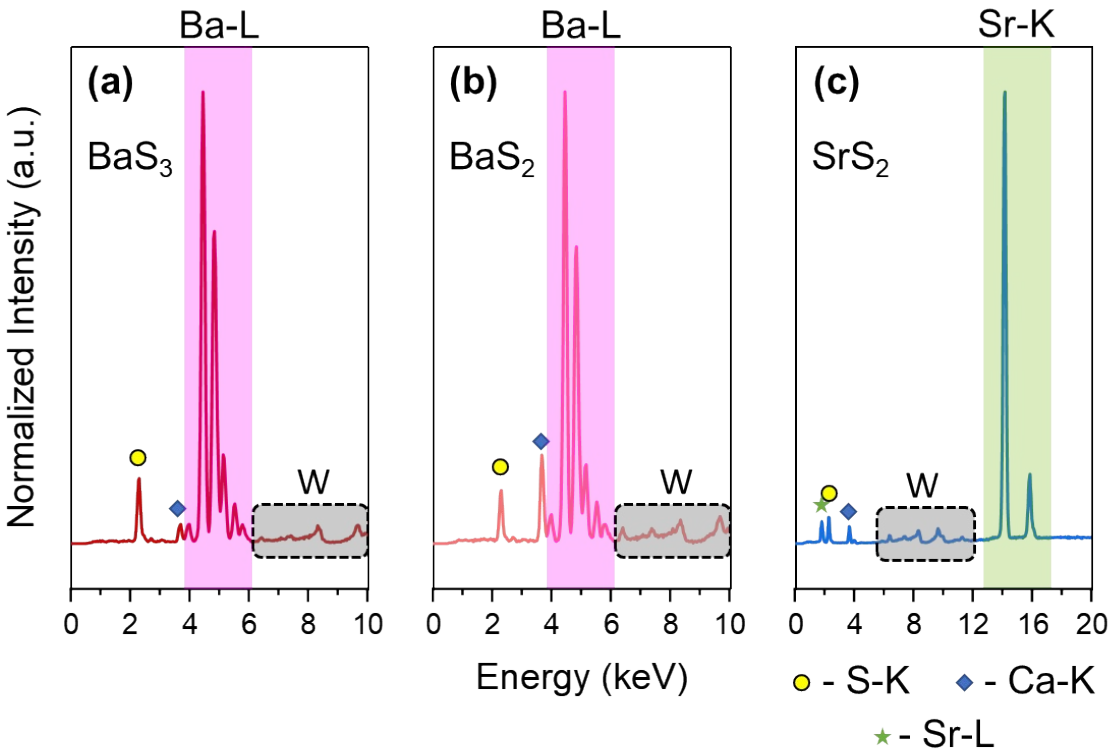

Figure S4. XRF spectra from the three polysulfides synthesized during this study. Shown in (a), (b), and (c) are the spectra measured for BaS_3, BaS_2, and SrS_2, respectively. Peaks marked with a yellow circle, blue diamond, and green star are from S-K, Ca-K, and Sr-L energies, respectively. The Ca signal is from the substrate (soda-lime glass) in which these samples were prepared and measured on. The peaks encircled with the gray box are W-M lines which are generated within the instrument optics.

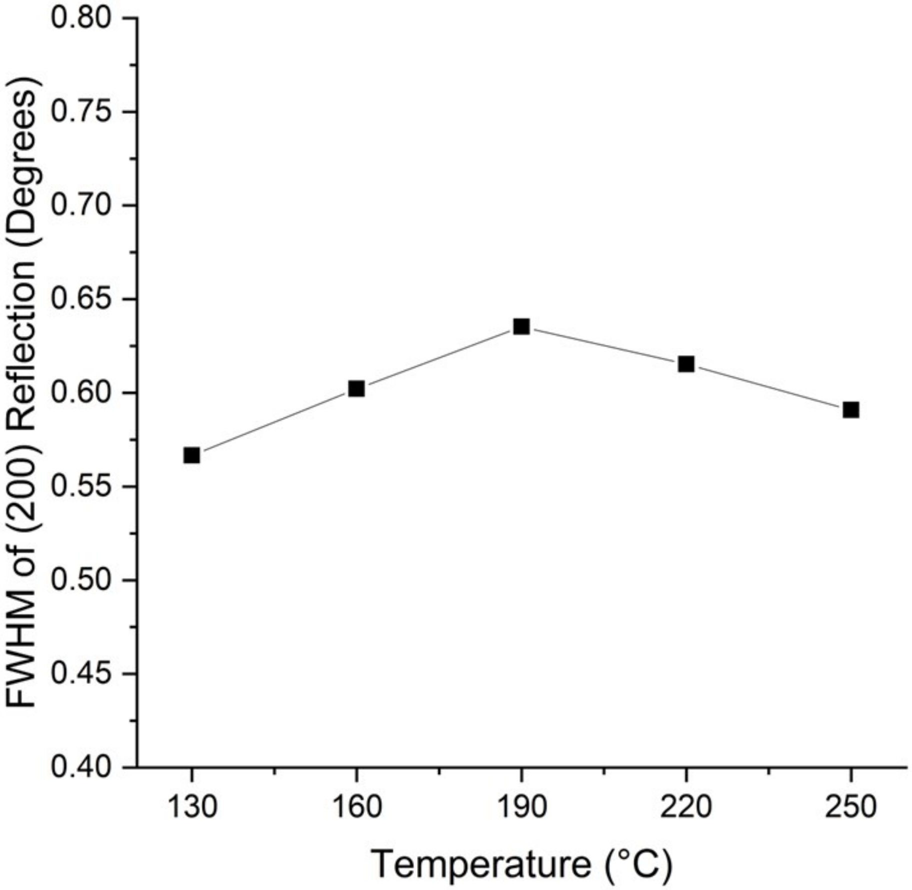

Figure S5. Additional data from the temperature study of the Ca-S system from 130 °C to 250 °C. No monotonic trend is observed for the FWHM values from the reflection caused by the (200) planes across the temperature range in this study. The 200 reflection is located at ~31.5° 2θ—the highest intensity reflection for CaS.

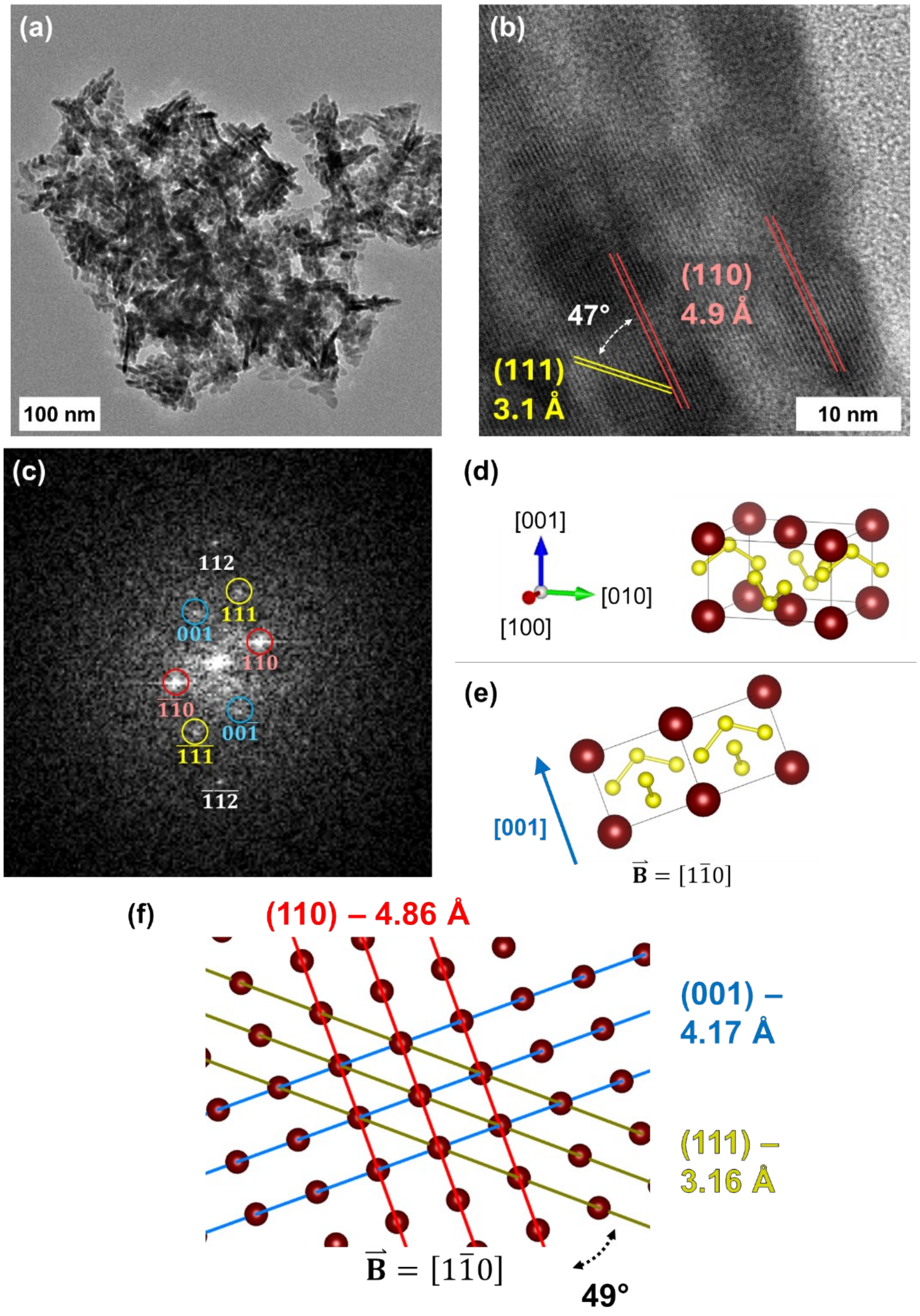

Figure S6. Additional TEM images of BaS_3 shown in (a) and (b) with identified lattice spacings in the latter. The FFT used to help identify lattice spacings is shown in (c). (d)-(f) provides illustrations from VESTA of the unit cell along with a BaS_3 lattice in (f) with crystal planes identified via the FFT. Here, barium atoms are shown in **dark red**, and sulfur atoms are **yellow**. (e) and (f) are illustrated from the $[1\bar{1}0]$ zone axis which is what the FFT in (c) was identified to be calculated from the image in (b). The planar spacings listed in (f) are those calculated from the BaS_3 standard (ICSD# 70059). Sulfur atoms are omitted from (f) for visual clarity.

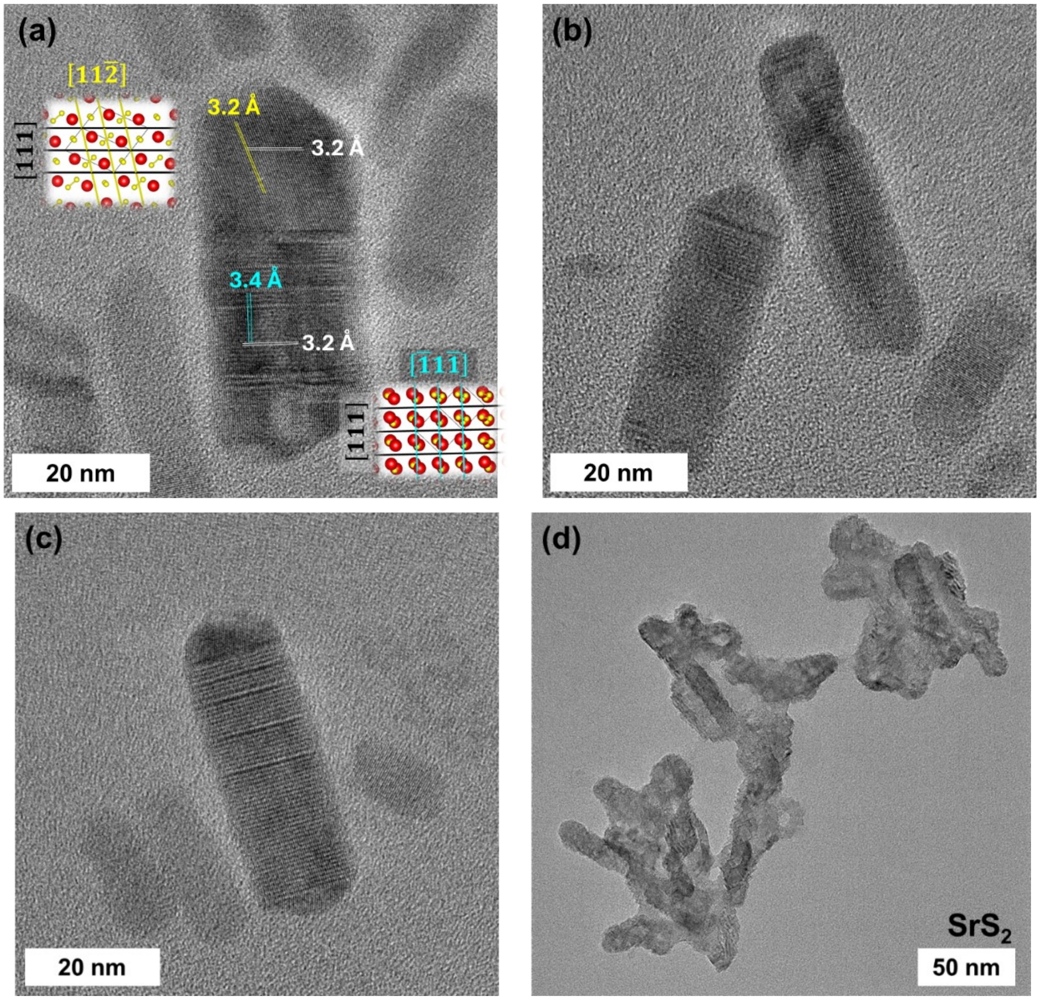

Figure S7. Additional TEM images of BaS_2 (a)-(c) and SrS_2 (d). Using the HRTEM image of the BaS_2 nanocrystal sample in (a), we attempted to correctly identify the lattice planes corresponding to the BaS_2 crystal structure. The $[11\bar{2}]$ plane has a calculated spacing of 3.26 Å, and the $[111]$ and $[\bar{1}1\bar{1}]$ planes both have a calculated spacing of 3.33 Å, which are the closest planar spacings in the BaS_2 standard (ICSD# 2004) to the spacings determined in these images. Also shown in (a) are insets of the BaS_2 crystal structure corresponding to our assigned crystal planes. Since the pXRD data shows no other major products beyond BaS_2, we believe that these apparent nanocrystal heterostructures may arise from the fact that BaS_2 has a high number of prominent crystal planes (shown by the high number of pXRD reflections) owing to its more complex crystal structure as a member of the monoclinic crystal system, which has many more degrees of freedom compared to a tetragonal crystal, like BaS_3. Many of these crystal planes also have very similar planar spacings (in addition to the ones already identified, i.e. the $\{310\}$, $\{202\}$, $\{40\bar{2}\}$,$\{31\bar{3}\}$, $\{021\}$, and $\{20\bar{4}\}$ crystal families which all lie in the ~38-40° 2θ and 2.36 to 2.25 Å ranges). These similarly sized crystal facets may also have similar formation energies potentially leading to this type of crystal growth, but further studies beyond the scope of this work, would be needed to investigate this. In addition to BaS_2, higher resolution images of the SrS_2 nanocrystals (d) seem to show a high degree of faceting at the nanocrystal surfaces.

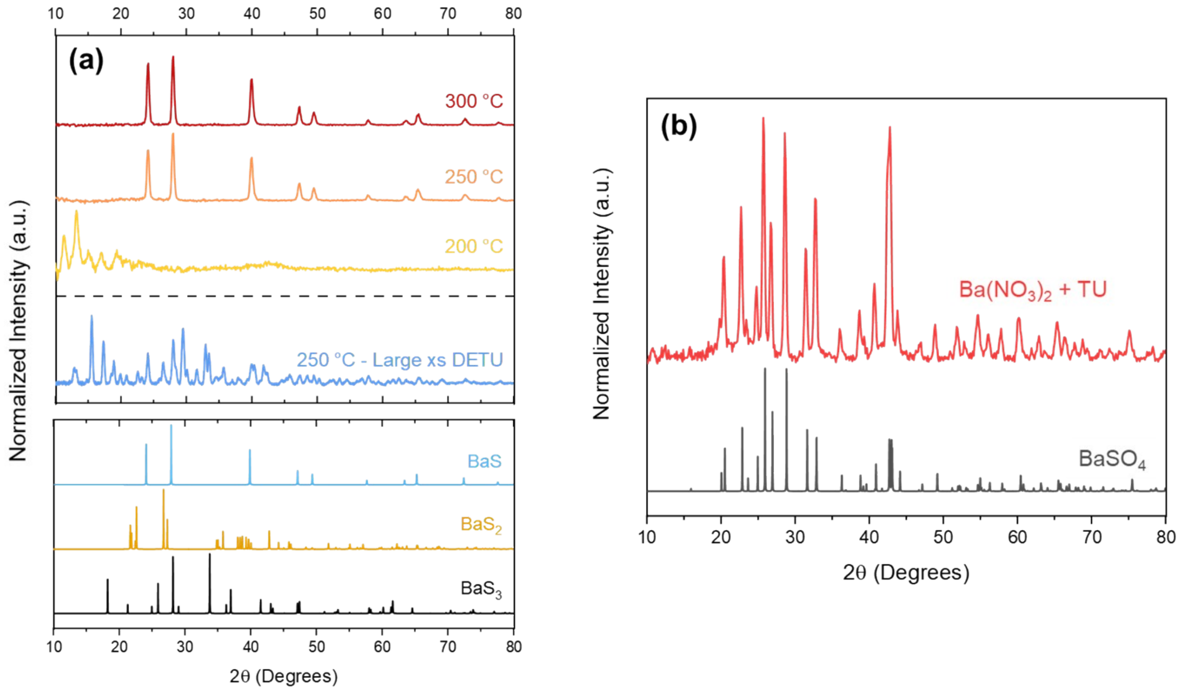

Figure S8. pXRD data from select experiments using different Barium precursors. $Ba(st)_2$ and TU (top three results) or 30x excess DETU (bottom result) were the precursors used in (a), and $Ba(NO_3)_2$ and TU were the precursors used in (b). The reaction in (b) was performed in *N*-methyl-2-pyrrolidone (NMP) rather than OLA. It should also be noted that the reactions performed in (a) were performed using a Schlenk line with Ar (non-sealed vessel), although we don't think it would have made a difference in these cases. More discussion is given below. The pXRD standards used are ICSD# 30240 (BaS), ICSD# 2004 (BaS_2), ICSD# 70059 (BaS_3), and ICSD# 33730 ($BaSO_4$).

Supplementary Discussion on Precursors Used

Barium stearate ($Ba(st)_2$) is a weakly reactive precursor as has been shown by Roth et al.,[1] needing higher temperatures than, say, $Ba(acac)_2$ for it to properly decompose to form the respective Ba chalcogenide. Hence, below 250 °C, we see many prominent peaks in the 200 °C sample using $Ba(st)_2$ + TU at low 2θ angles, presumably belonging to unreacted $Ba(st)_2$ and/or partially decomposed byproducts. In addition to weakly reacting precursors, Barium also has many salts that are only slightly soluble to insoluble in organic solvents, such as $Ba(NO_3)_2$ and $Ba(OAc)_2$ limiting their applications towards this protocol. When reacted with S in OLA for 30 min at 250 °C, reactions with both precursors visually showed that no reaction had proceeded (no colloid formation—data not shown). Both $Ba(NO_3)_2$ and $Ba(OAc)_2$ appear to have very little to no solubility in OLA which likely prevented any significant reaction from occurring. Upon trying a

more polar reaction solvent (NMP) and the substitution of S with TU, a reaction did occur when using $Ba(NO_3)_2$, but the resulting major product was shown to be $BaSO_4$ (**Figure S8b**). TU was used in place of S due to its higher solubility in polar solvents and its ability to form an organo-chalcogen complex with metal ions, increasing the solubility of precursors used.[2,3]

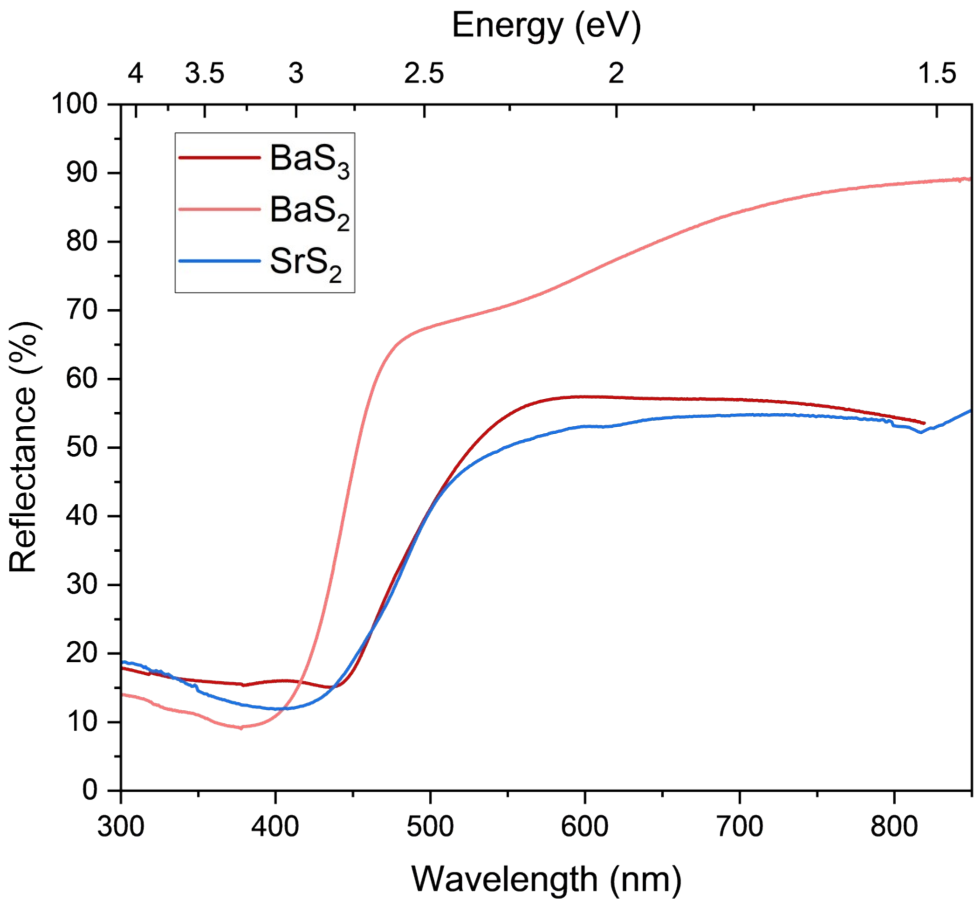

Figure S9. Raw diffuse reflectance spectra of the BaS_3, BaS_2, and SrS_2 samples. These are the same samples shown in the data presented in **Figure 5** from the main text. These measurements were performed on nanocrystals synthesized at 160 °C, 250 °C, and 160 °C for BaS_3, BaS_2, and SrS_2, respectively.

Supplementary References:

(1) Roth, A. N.; Chen, Y.; Adamson, M. A. S.; Gi, E.; Wagner, M.; Rossini, A. J.; Vela, J. Alkaline-Earth Chalcogenide Nanocrystals: Solution-Phase Synthesis, Surface Chemistry, and Stability. *ACS Nano* **2022**, *16* (8), 12024–12035. https://doi.org/10.1021/acsnano.2c02116.

(2) Tan, J.; Zhang, X.; Suh, J.; Ha, N.; Lee, J.; Tilley, S. D.; Yang, W. Molecular Ink-Derived Chalcogenide Thin Films: Solution-Phase Mechanisms and Solar Energy Conversion Applications. *Mater Today Energy* **2023**, *34*, 101288. https://doi.org/10.1016/j.mtener.2023.101288.

(3) Clark, J. A.; Murray, A.; Lee, J.; Autrey, T. S.; Collord, A. D.; Hillhouse, H. W. Complexation Chemistry in N,N -Dimethylformamide-Based Molecular Inks for Chalcogenide Semiconductors and Photovoltaic Devices. *J Am Chem Soc* **2019**, *141* (1), 298–308. https://doi.org/10.1021/jacs.8b09966.
